# Immunotherapy as a Precision Medicine Tool for the Treatment of Prostate Cancer

**DOI:** 10.3390/cancers13020173

**Published:** 2021-01-06

**Authors:** Maria Adamaki, Vassilios Zoumpourlis

**Affiliations:** Biomedical Applications Unit, Institute of Chemical Biology, National Hellenic Research Foundation (NHRF), 48 Vassileos Constantinou Avenue, 11635 Athens, Greece; vzub@eie.gr

**Keywords:** prostate cancer, immunotherapy, precision medicine, predictive biomarkers, immune checkpoint inhibitors

## Abstract

**Simple Summary:**

Immunotherapy has emerged as an attractive alternative to conventional therapies for the clinical management of prostate cancer (PCa); it not only specifically targets malignant cells while protecting healthy tissue, but it also appears to augment the therapeutic effect of existing treatments when used in combination. The identification of biomarkers with prognostic and preventive clinical significance has facilitated the incorporation of immune-targeted agents in clinical trials, with the aim to assess therapeutic efficacy in patient sub-populations that have been stratified on the basis of specific molecular traits and prognostic variables. This perfectly fits the rationale of precision medicine, which aims to match patients with targeted therapies so as to achieve the maximum clinical benefit. The numerous clinical trials currently evaluating multiple immunotherapeutic approaches in PCa patients, both alone and in combination with other treatments, offer much hope for achieving significant advances in the decision for precision treatment of the disease.

**Abstract:**

Prostate cancer (PCa) is the most frequently diagnosed type of cancer among Caucasian males over the age of 60 and is characterized by remarkable heterogeneity and clinical behavior, ranging from decades of indolence to highly lethal disease. Despite the significant progress in PCa systemic therapy, therapeutic response is usually transient, and invasive disease is associated with high mortality rates. Immunotherapy has emerged as an efficacious and non-toxic treatment alternative that perfectly fits the rationale of precision medicine, as it aims to treat patients on the basis of patient-specific, immune-targeted molecular traits, so as to achieve the maximum clinical benefit. Antibodies acting as immune checkpoint inhibitors and vaccines entailing tumor-specific antigens seem to be the most promising immunotherapeutic strategies in offering a significant survival advantage. Even though patients with localized disease and favorable prognostic characteristics seem to be the ones that markedly benefit from such interventions, there is substantial evidence to suggest that the survival benefit may also be extended to patients with more advanced disease. The identification of biomarkers that can be immunologically targeted in patients with disease progression is potentially amenable in this process and in achieving significant advances in the decision for precision treatment of PCa.

## 1. Introduction

Prostate cancer (PCa), an age-related disease predominantly affecting men over the age of 60, is the most frequently diagnosed type of cancer and the second most common cause of cancer-related death, after skin cancer, among men worldwide [1,2]. The disease is characterized by remarkable heterogeneity, and patients with apparently similar histological features usually display a variety of clinical behavior and outcome, ranging from decades of indolence to highly lethal disease [3]. This is probably the reason behind the observed substantial mortality from aggressive disease, despite the majority of patients being diagnosed with slow-progressing or even inert PCa [2]. The disease has a greater prevalence in the West [4,5], yet considerable variability exists among certain populations; men of African ancestry appear more susceptible to developing PCa and have a worse prognosis than white men or men of Hispanic origin [6,7] whereas Hispanic men exhibit significantly lower incidence and mortality rates than non-Hispanic white men [8]. In addition to age and race, a family history also increases the risk of developing the disease by even two- to three-fold if the affected individual is a first-degree relative [9], thereby ranking PCa among the cancers with the highest heritability [10,11]. On the other hand, migrant studies have found that populations of the same race and origin may increase their risk of developing PCa over time by moving to countries with a higher incidence of the disease [12]; this suggests that, apart from genetic contributors, lifestyle, and environmental factors are also actively involved in the development of the disease. Such factors may include a diet high in red meat, milk products, processed food, fat content, and low in fruit and vegetables [9], as well as tobacco use, obesity, and lack of physical activity [12]. 

Therapeutic options range from active surveillance in cases of less aggressive disease, to radiation therapy for localized disease, and surgery in combination with cytotoxic therapy for more advanced disease. If the cancer is limited to the prostate, then it is described as localized disease and considered curable; if it has spread outside the prostate to the bones or other sites, then several targeted therapies can be used, including hormonal treatment, chemotherapy, radiotherapy, and immunotherapy [13,14]. Clinical outcome is significantly associated with age, underlying health conditions, cancer histology, and the extent of disease [15]. Suppression of androgen receptor (AR) signaling through androgen deprivation therapy (ADT) has been the primary therapeutic approach for metastatic PCa for more than 70 years, since its benefits were first reported by Charles Huggins in 1941 [16,17]. Nowadays, this translates to either surgical or medical castration, the latter including the use of luteinizing hormone-releasing hormone agonists or antagonists, regardless of whether anti-androgen drugs are being used or not [16]. Despite the high rate of progression-free survival (PFS) following ADT, with near-certain remissions usually lasting 1–2 years in the majority of cases, 30–50% of patients progress to castration-resistant prostate cancer (CRPC) and eventually relapse [18]. CRPC includes the spectrum of PCa ranging from asymptomatic disease to advanced CRPC (metastatic CRPC or mCRPC), characterized by an over-activation and over-expression of the AR, which results in the transcription of downstream target genes that promote carcinogenesis [19,20]. In patients with mCRPC the cancer cells usually spread to the bones and lymph nodes [21], ultimately developing therapeutic resistance regardless of the treatment modality applied, whether this is anti-androgen therapy, cytotoxic drugs, or radiopharmaceuticals. These patients have limited treatment options and a very bad prognosis [22]. Metastatic bone disease, in particular, is the main cause of PCa-related pain requiring palliative radiotherapy and of serious skeletal-related events such as bone fractures and spinal cord compression, often requiring orthopedic surgery, which greatly influence patient quality of life [23]. Considering that approximately one-fifth of the world’s population is estimated to be ≥ 60 years old by the year 2050 [22], this also highlights the profound socio-economic consequences of the disease and the urgency for devising new therapies.

Since the completion of the TAX327 trial in 2004, docetaxel plus prednisolone has been established as the first-line chemotherapeutic treatment for CRPC, offering a modest 2.5-month prolongation of median overall survival (OS) before the emergence of therapeutic resistance [16,24,25]. In the last decade or so, the development of new technologies for the characterization of PCa has led to significant progress in the field of systemic treatment and to the approval of additional drugs. These include the chemotherapeutic drug cabazitaxel [26], the androgen signaling inhibitors abiraterone, enzalutamide, apalutamide, and darolutamide [27,28,29], the alpha-emitter bone-seeking radioisotope radium-233 [30,31], and the immunotherapeutic drug Sipuleucel-T [32], all of which have demonstrated significant improvements in OS. However, despite the development of these relatively rapid therapeutic interventions, and the testing of many more such compounds in clinical trials, approved therapies are administered to relatively unselected patients, solely based on clinical characteristics such as performance status and tolerance [3]. Optimal sequencing and combinations of drugs are yet to be determined, as is also the selection of reliable biomarkers for predicting response to therapy. In this context, precision medicine aims to implement rational combination treatment schemes and to match patients with targeted therapies so as to optimize therapeutic effects and to prevent metastatic disease. Considering the remarkable heterogeneity of PCa, its molecular complexity, and its multifactorial nature, the incorporation of clinically valuable prognostic and predictive biomarker stratification for appropriate patient selection is potentially amenable in this process. In addition, the management of PCa is beginning to embrace the precision medicine approach with the use of new technologies, such as liquid tumor profiling, non-coding RNA diagnostics, genomic and proteomic analysis, gene editing, array-based technologies, and next-generation sequencing [2,3,33].

In the past few decades, significant advances have also been achieved in our understanding of the immune system and its relationship with cancer. As the immune system is able to recognize and eliminate newly developing cancer cells, and therefore capable of preventing the onset and progression of malignant disease [34], immunotherapy has emerged as a promising treatment modality for a number of cancer types, including melanoma, renal cell carcinoma, hematologic malignancies, breast cancer and PCa [35]. By targeting malignant cells while at the same time sparing healthy tissue from the damage that is usually induced by radiation and chemotherapy, immunotherapy offers the promise of a non-toxic and efficacious treatment alternative [36]. PCa has attracted a lot of interest as a suitable target for immunotherapeutic intervention, mainly because the tissue itself expresses multiple tumor-associated antigens (TAAs) which the adaptive immune system recognizes, and is also characterized by relatively slow growth kinetics which may provide a longer time frame for the development of effective anti-tumor immune responses [37]. Even though Sipuleucel-T is currently the only FDA-approved immunotherapy option for PCa, with demonstrated PFS (progression-free survival) or OS improvement in clinical trials, there is ample promise on the horizon, as a large number of clinical trials are evaluating various immunotherapeutic approaches in PCa patients; these include immune checkpoint inhibitors, tumor-specific antigen approaches in the form of vaccines, and immunomodulating agents such as antibodies and antibody-drug conjugates [38]. 

This review gives a detailed account of the immunologic platforms that have so far been associated with immunotherapeutic efficacy and which may constitute crucial targets for achieving significant advances in the decision for precision treatment of PCa. We discuss therapeutic efficacy in patient sub-populations that have been stratified on the basis of prognostic variables and highlight the patient groups most likely to benefit from immunotherapeutic interventions. Finally, we describe the clinical barriers associated with the application of immunotherapy in the management of the disease, as well as possible solutions to circumventing these problems.

## 2. Immunotherapy as a Precision Treatment Tool for PCa

Disease occurrence and progression in prostate cancer are regarded as a function of biomarkers, mainly in the form of tumor-specific antigens or genetic aberrations that can be used for diagnosis, risk assessment, and prognosis, as well as for precision-guided therapeutics [39]. Diagnostic biomarkers for PCa are essentially prostate-specific antigens with the potential to not only discriminate between indolent and advanced disease, but also to be targeted therapeutically; these include prostate-specific-antigen (PSA), prostate acid phosphatase (PAP), prostate-specific membrane antigen (PSMA), prostate stem cell antigen (PSCA), prostate cancer antigen 3 (PCA3), NY-ESO-1, mucin-1 (MUC1), GRB2-like endophilin B2 (SH3GLB2), T-cell receptor alternate reading frame protein (TARP) and the six transmembrane epithelial antigens of the prostate (STEAP), among many others [40,41]. During the last decade, there has been a significant increase in the number of identified prostate-specific genomic biomarkers that can be used to reliably estimate relative genetic risk, prognosis and tumor aggressiveness of the disease, and have therefore been the subject of intense investigation for their significance in the decision for therapy selection [42,43,44,45]. Importantly, PCa biomarkers can also become molecular targets for immunotherapy: diagnostic biomarkers because they constitute prostate-specific antigens that the immune system can be primed to recognize, and genomic biomarkers because they may include genes that are involved in the regulation of the immune response [43]. 

**Figure 1:**
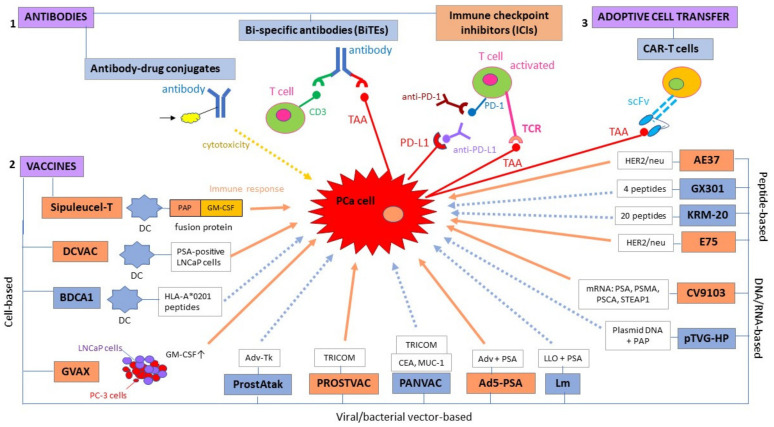
Immunotherapeutic strategies for prostate cancer fall into three main categories: (1) antibodies, (2) vaccines, and (3) adoptive cell transfer; these can be subdivided into smaller categories depending on the mode of action. Immunotherapeutic modalities in orange boxes represent strategies that have been shown to confer a survival advantage to prostate cancer (PCa) patients, whereas immunotherapies in blue boxes are either in pre-clinical/early clinical development or they have so far failed to demonstrate a survival benefit in terms of progression-free survival (PFS) or overall survival (OS). Similarly, orange arrows represent an immune response, whereas dotted blue arrows represent a possible but not yet confirmed immune response. Ad5: adenovirus type 5; AdV-tk: adenoviral vector containing a herpes virus-derived thymidine-kinase; CEA: carcinoembryonic antigen; DC: dendritic cell; GM-CSF: granulocyte-macrophage colony-stimulating factor; HLA: human leukocyte antigen; Lm: listeria monocytogenes; LLO: listeria monocytogenes (Lm)-listeriolysin O; LNCaP: lymph node-derived human prostate adenocarcinoma cell line; MUC-1: mucin-1; PAP: prostatic acid phosphatase; PC-3: prostate cancer cell line derived from bone metastasis; PD-1: programmed death receptor-1; PD-L1: programmed death-ligand 1; PSCA: prostate stem cell antigen; PSMA: prostate-specific membrane antigen; scFv: single chain variable fragment; STEAP: six transmembrane epithelial antigen of the prostate; TRICOM: TRIad of Co-stimulatory Molecules.

Immunotherapies for PCa include both passive treatment approaches, such as direct delivery of monoclonal antibodies (mAbs) with high tumor antigen specificity, and active approaches, such as vaccines. Adjuvants for cancer immunotherapy include organic molecules, inorganic compounds, nanoparticles, polymers, and colloids such as gels, sols, and emulsions, and can be combined with both active and passive forms of immunotherapy with the aim to enhance the immune response [46,47]. Notably, cytokines can be used as adjuvants in combination with other immunotherapeutic agents, as, for example, in tumor cell-based cancer vaccines [36]. Immunotherapeutic strategies employing monoclonal antibodies can be further divided into antibody-drug conjugates [48,49,50,51,52], artificial bi-specific T cell-engaging antibodies, or BiTEs [53,54,55,56,57,58], and immune checkpoint inhibitors [59]. Cell-based immunotherapy, on the other hand, is the adoptive cell transfer (ACT) into patients of T cells that have been genetically modified to contain a chimeric antigen receptor (hence the term CAR T cells) that targets a prostate-specific antigen [59,60,61]; nonetheless, some of these immunotherapeutic interventions are still in the pre-clinical or early clinical (phase I) stage and, as such, they do not yet provide enough evidence to support therapeutic efficacy (Figure 1). Below, we discuss the immunotherapeutic strategies that have been shown to confer clinical benefit to PCa patients, in terms of PFS or OS, and which may be further investigated as precision treatment options for PCa. 

### 2.1. Immune Checkpoint Inhibitors

Immune checkpoint inhibitors (ICIs) represent a class of mAbs that have the ability to inhibit immune checkpoint receptors and, therefore, to prevent the inactivation of T-cell function. Immune checkpoint receptors include cytotoxic T lymphocyte-associated protein 4 (CTLA-4), programmed death 1 (PD-1), and programmed death ligand 1 (PD-L1), and antibodies (ICIs) against them have been shown to induce potent anti-tumor immune responses in a variety of cancers [59].

CTLA-4 is a transmembrane protein that is expressed on T lymphocytes and competitively binds CD80 and CD86 on antigen-presenting cells (APCs), thereby creating a negative feedback loop that prevents T-cells from killing other cells, including cancer cells [62]. Ipilimumab, the ICI blocking the function of CTLA-4, started being tested in PCa clinical trials shortly after its FDA approval for the treatment of melanoma in 2011 [63,64]. Following encouraging results from phase I studies in patients with mCRPC, where it was shown that ipilimumab in combination with granulocyte macrophage-colony-stimulating factor (GM-CSF) in PCa vaccines induces significant PSA declines, an open-label phase I/II multicenter study (NCT00323882) investigating ipilimumab in patients with mCRPC, suggested clinical anti-tumor activity, as supported by manageable adverse events and substantial disease control [65,66,67]. A subsequent phase III clinical trial (NCT00861614) found a significantly higher OS in mCRPC patients with favorable prognostic characteristics that received ipilimumab as compared to a placebo drug [59,68], with OS rates following a two to three times higher trend at three years onwards in cases where ipilimumab was administered along with radiotherapy [69]. On the contrary, patients with asymptomatic or minimally symptomatic chemotherapy-naïve PCa have not been shown to gain any clinical benefit from ipilimumab monotherapy in terms of OS [70]. 

PD-1 is another transmembrane protein expressed on T cells; its receptor interacts with the PD-1 ligand (PD-L1) that is expressed on both normal and malignant cells, and their interaction constitutes an important checkpoint of T lymphocyte inhibition [62,71]. PD-1 binding to PD-L1 on tumor cells results in an inhibition of apoptosis, T-lymphocyte tolerance and an increase in tumor cell survival [71]. ICIs that act as inhibitors of PD-1 include nivolumab and pembrolizumab, whereas ICIs of PD-L1 include atezolizumab, avelumab, and durvalumab [62]. Even though primary prostate cancers are characterized by an infiltration of PD-1 expressing CD8+ T cells, mCRPC shows minimal expression of the PD-L1 ligand, which represents a significant obstacle when applying anti-PD-1/PD-L1 monotherapy [59,72,73]; only mCRPC patients who have developed resistance to the anti-androgen enzalutamide have been associated with an upregulation of PD-L1 [74]. Clinical trials testing nivolumab or pembrolizumab as monotherapy in unselected mCRPC patients have produced unsatisfactory results in terms of demonstrating a significant survival benefit, with the only exceptions being partial responses in enzalutamide-resistant patients and in patients with microsatellite instability (MSI) [72,75,76]. Indeed, there is enough evidence to suggest that patients with DNA mismatch repair mechanism (MRM) mutations demonstrate anecdotal sensitivity and may derive benefit from treatment with pembrolizumab, possibly due to the higher rate of TAAs and MSI, despite these being relatively uncommon in PCa [62,76,77,78]. Preliminary data from recent clinical trials seem to be more encouraging, as pembrolizumab monotherapy has been shown (i) to induce durable objective responses and minimal adverse events in a subset of patients with heavily pre-treated, advanced PD-L1-positive PCa (NCT02054806) [79] and (ii) to evoke anti-tumor activity with durable clinical responses and significant OS estimates in a subset of treatment-refractory (docetaxel and one or more targeted endocrine therapies) mCRPC patients (NCT02787005) [80]. Therefore, PD-L1 expression, high MSI and high tumor mutational burden (TMB) are currently regarded as biomarkers for pembrolizumab therapy selection for patients with advanced PCa [81].

However, despite the promising results of pre-clinical and clinical studies, immune checkpoint blockade-based immunotherapy appears to be less efficient in PCa as compared to other cancer types; this is mostly due to the fact that prostate cancer is a “cold” tumor, characterized by minimal T-cell infiltration, low TMB, low PD-1 expression and downregulated or non-existent MHC class I expression, a necessary prerequisite for successful immune checkpoint inhibition [62]. In order to overcome therapeutic limitations, to enhance the therapeutic potential of ICIs and to extend the survival benefit to larger subsets of patients, many studies are currently investigating combinations of two different types of ICIs, or ICIs with other treatment modalities. Examples include combination regimens of nivolumab with ipilimumab [82,83,84], nivolumab with docetaxel [85], pembrolizumab with enzalutamide [86,87], and ipilimumab with GM-CSF [65], among many others. A significant number of clinical trials are currently ongoing or recruiting with the aim to investigate the efficacies of ICIS, both in monotherapy and in various therapeutic combinations; a list of ongoing studies is given in Table 1.

### 2.2. Vaccines

Therapeutic vaccines for PCa are designed with the aim to elicit an adaptive immune response via antigen presentation; because prostate cells express remarkably more TAAs than most other types of human tissue, TAAs constitute the most significant part, followed by immune adjuvants such as cytokines [62]. The major categories that have so far been shown to offer some clinical advantage in terms of prolonged PFS or OS are as follows:

#### 2.2.1. Cell-Based Vaccines

Cell-based vaccines can be immune cell-based, such as dendritic cell vaccines, or whole tumor cell-based, hence the name tumor cell vaccines. 

Sipuleucel-T is an autologous dendritic cell (DC) vaccine that elicits an immune response to the prostatic acid phosphatase (PAP) antigen; vaccine preparation includes leukapheresis of peripheral mononuclear cells from the patient, their ex-vivo exposure to a fusion protein containing the PAP antigen and GM-CSF for 36–44 h and then infusion back into the patient for a total of three treatments over a 6-week period [32,104]. It is currently the only FDA-approved therapeutic cancer vaccine and is specifically aimed at asymptomatic or minimally symptomatic mCRPC patients with no visceral metastases, following evidence of improved OS in the phase III setting of four clinical trials, namely D9901 (NCT00005947) [105], D9902A (NCT01133704) [106], IMPACT (NCT00065442) [32] and PROTECT (NCT00779402) [107]. The extended survival benefit was estimated at approximately 4 months, as compared to the placebo group, which, combined with no measurable change in PSA or tumor burden and no significant difference in disease progression, caused enough controversy regarding the utility of sipuleucel-T in the management of PCa [32,105,106,108]. However, it was later realized that the survival effect might have been underestimated, as patients in the placebo group with disease progression, who constituted over 50% of the total patients enrolled in the IMPACT study [32], were allowed to cross over to receive the vaccine; these patients had a significantly longer OS of 20 months, as compared to the 9.8 months in the group that never crossed over [105,109]. Consistent with these findings, a more recent trial, named PROCEED (NCT01306890), has provided further evidence of Sipuleucel-T’s safety and tolerability, and of a longer OS benefit in lower baseline PSA quartiles as opposed to higher baseline PSA quartiles [109,110,111]. Importantly, a substantial number of patients experienced a long treatment-free interval between Sipuleucel-T and subsequent therapeutic regimens, which may also reflect a clinical benefit [111]. On the contrary, sipuleucel-T is not supported for use in CRPC patients with only increased serum PSA levels as evidence of disseminated disease [107,109,112,113], but there is enough evidence to suggest that patients with localized PCa before radical prostatectomy might benefit from a systemic and local tumor response to vaccine treatment [62,114,115].

DCVAC/PCa is another dendritic cell vaccine involving leukapheresis and in vitro activation of autologous mature dendritic cells pulsed with killed PSA-positive LNCaP cells [116]. Phase II clinical trials testing the efficacy of the vaccine have produced conflicting results. For example, one study evaluating DCVAC/PCa in combination with prednisone and docetaxel chemotherapy in men with mCRPC has demonstrated a manageable safety profile, a 6–7 month OS advantage and the induction and maintenance of PSA-specific T cells [88,117]; another study did not find this combination regimen to be beneficial in the long term, despite the induction of an immune response [105,109,118]. As a result, a randomized, double-blind, placebo-controlled, phase III clinical trial (VIABLE, NCT02111577) is currently ongoing with the aim to determine the safety and efficacy of DCVAC/PCa in combination with docetaxel and prednisone (versus docetaxel and prednisone alone) in patients with mCRPC [119].

GVAX is a tumor cell-based vaccine which, as the name implies, consists of irradiated whole tumor cells that have been genetically modified to constitutively express GM-CSF; the prostate tumor cells are extracted from the hormone-sensitive cell line LNCaP and the hormone-refractory cell line PC-3, which derive from nodal and bone sites of metastasis, respectively [88,120]. This strategy has the advantage of inducing immune responses to multiple TAAs without the need for HLA matching [121]. So far, an enhanced median survival has only been observed in hormone-refractory PCa patients following high dose boost vaccinations as compared to low dose boosts and radiotherapy in phase I/II settings [122,123,124].

#### 2.2.2. Vector-Based Vaccines 

Vector-based vaccines may include vectors derived from oncolytic viruses, based on the rationale that these can infect tumor cells and cause them to self-destruct, or vectors derived from bacterial pathogens that are actively phagocytosed by APCs and are thereby able to generate TAAs and to enable specific T cell immune responses.

PROSTVAC-VF (PSA-TRICOM) is a viral vector vaccine that uses two recombinant poxvirus vectors, both of which include a plasmid carrying the transgenes that code for PSA: one that is derived from vaccinia virus (PROSTVAC-V) and contains a triad of T cell co-stimulatory molecules (TRICOM), namely LFA-3, B7.1 and ICAM-1, in conjunction with PSA, and one that is derived from fowlpox virus (PROSTVAC-F) which serves to deliver booster doses [20,62,109]. The scientific rationale behind this vaccine is that the vaccinia vector acts as a single dose immunogenic factor, eliciting a strong immune response both against PSA and the viral protein, leading to the destruction of PSA-positive tumor cells and subsequently to the release of a wider range of TAAs (antigen spreading) that stimulate additional pro-inflammatory signals and additional tumor-specific T cell immune responses [109,125,126]. Under the same rationale, the fowlpox vector, transduced to code for the same TAA (PSA), is used for subsequent booster vaccinations in order to bypass the problem of the vaccinia virus vector being neutralized by the host immune system, as the former (PROSTVAC-F) is able to penetrate APCs without invoking the production of high volumes of neutralizing antibodies [62,127,128]. 

PROSTVAC-VF clinical testing in patients with localized PCa and in patients with advanced PCa has produced inconclusive results in terms of demonstrating an improvement in PFS or OS [62,129,130,131,132,133,134,135,136]. However, when used in combination with other forms of PCa therapy, such as chemotherapy, ADT, radiotherapy, and ICIs, the immunotherapeutic efficacy of the vaccine seems to be endorsed [62,109]. For example, concurrent administration of PROSTVAC-VF with docetaxel in metastatic androgen-independent PCa patients has been shown to confer a longer PFS as compared to patients receiving chemotherapy alone [137]. Similarly, patients with non-metastatic CRPC who receive the vaccine prior to second-line anti-androgen therapy with nilutamide may derive a greater clinical benefit in terms of improved OS, as compared to patients who receive nilutamide alone or prior to immunotherapy [138,139]; preliminary evidence from a randomized phase II study investigating the efficacy of the anti-androgen flutamide with and without PROSTVAC-VF in patients with non-metastatic CRPC suggests an improvement in time to treatment failure in the combination arm [140]. Interestingly, the concurrent administration of PROSTVAC-VF and the radiopharmaceutical samarium-153-EDTMP in patients with non-visceral mCRPC has demonstrated a PSA response and longer PFS in the combination arm [62,141]. As a result, PROSTVAC-VF is currently being investigated in early phase trials as combination therapy with ICIs such as nivolumab (NCT02933255) in patients with localized PCa and CRPC, with nivolumab/ipilimumab (NCT03532217) in patients with metastatic, hormone-sensitive PCa, as well as in conjunction with other immunotherapeutic agents such as bi-functional fusion protein MSB011359C (targeting PD-L1 and TGF-β) in men with recurrent disease after localized radical treatment [109,142,143]. 

Ad5-PSA is another viral-vector vaccine derived from replication-deficient recombinant adenovirus type 5 (Ad5), based on pre-clinical evidence that the Ad5 vector has the ability to elicit durable cellular and humoral immune responses, especially when combined with a gelfoam collagen matrix that acts as an adjuvant, even in the presence of high titer anti-adenovirus antibodies [144,145]. Substantial anti-PSA immune responses and prolonged survival have been observed in patients with measurable mCRPC in a phase I clinical setting [146], whereas preliminary results from a phase II trial (NCT00583024) investigating Ad5-PSA diluted in gelfoam matrix in patients with non-metastatic and early metastatic CRPC suggest a prolonged metastasis-free survival benefit [147]. Interestingly, chimpanzee adenoviral (ChAd) vectors might represent an attractive alternative to human Ad5 vectors as vaccine candidates for PCa immunotherapy, as recent evidence not only highlights their safety and ability to induce antigen-specific humoral and cellular immunity, but also their ability to bypass the problem of pre-existing immunity that is associated with human Ad vectors [148].

#### 2.2.3. DNA/mRNA-Based Vaccines

DNA- and RNA-based vaccines consist of plasmid DNA and mRNA, respectively, that is modified to encode for a tumor-specific antigen, resulting in an immune response when expressed by transfected cells via both the MHC class I and MHC class II pathways [149,150]. They represent a promising alternative to conventional vaccine strategies due to their high potency, developmental feasibility, low manufacturing cost, and acceptable safety profile [150].

CV9103 is an mRNA-based vaccine encoding for a number of different TAAs simultaneously: PSA, PSMA, PSCA, and STEAP1 [109,151]. A phase I/II study investigating CV9103 in patients with CRPC with rising PSA and predominantly existing metastases declared that the vaccine is safe, well-tolerated and displays a remarkably high level of cellular immunogenicity [152]; subsequent analysis revealed that a significant correlation exists between immunogenicity and a prolonged survival outcome, despite unfavorable patient characteristics, thereby suggesting a therapeutic benefit [153]. 

#### 2.2.4. Antigen or Peptide-Based Vaccines

Based on the rationale that individual patients will elicit substantially different immune responses against TAAs, due to both their tumor and immune cells being diverse and heterogeneous, personalized selection and administration of HLA-matched peptides, based on pre-existing patient immunity status prior to vaccination, constitutes an attractive immunotherapeutic approach referred to as personalized peptide vaccination (PPV) [154]. Pre-existing host immunity, or immunological memory, to the vaccine antigen(s) is a necessary prerequisite in order to induce rapid and robust immune responses [155]. The advantage of peptide-based vaccines is that peptides induce robust and rapid cytotoxic T lymphocyte (CTL) activation without the costs and cell availability limitations associated with cell-based vaccines. In this respect, many vaccine TAA peptides have so far been identified, both for CTL (MHC class I) and for T helper cells (MHC class II), with numerous platforms investigating PPVs both as monotherapy and in combination with other forms of cancer therapy [156]. Below we discuss the most promising immunotherapeutic platforms employing PPV.

PPV plus chemotherapy: Significantly longer PFS and OS have been observed in HLA-A24-positive CRPC patients treated with 14 PPVs in combination with low-dose estramustine phosphate (EMP), a dual estrogen and chemotherapy medication (nornitrogen mustard linked to estradiol-1β-phosphate), as compared to standard dose EMP, with peptide-specific immune responses also being strongly associated with PSA doubling time [157,158,159]. 

PPV plus glucocorticoids: Following substantial evidence that low doses of dexamethasone can be beneficial in the treatment of hormone-refractory PCa, both as monotherapy and in combination with PPV [160,161,162,163,164], a clinical benefit has also been demonstrated in chemotherapy-naïve CRPC patients receiving PPV immunotherapy with low-dose dexamethasone (as compared to receiving dexamethasone alone), evidenced as both longer PSA-related PFS and OS [165]. 

HER2/neu peptides: these constitute TAAs that appear to be over-expressed in a variety of cancers, including PCa, and have therefore been used as targets of active immunotherapy [166,167]. Specifically, HER-2/neu has been shown to stimulate cell division and to activate the AR pathway in the absence of androgen, thereby increasing the malignant potential of prostate cancer cells and the development of CRPC [168,169,170,171]. One such example is E75 (HER2/neu 366–379, or nelipepimut-S), a nine amino acid peptide derived from the extracellular domain of the HER2 protein that has been shown to elicit prominent immunologic responses in both the pre-clinical and clinical settings [172]. Specifically, cell cultures from PCa patients at risk of recurrence stimulated with E75 appeared to activate E75-specific lymphocytes with tumor-specific cytolytic activity against the HER2/neu-positive cell lines [173]. Similarly, in a phase I/II trial investigating the safety and efficacy of E75 in preventing PSA recurrence in high-risk PCa patients, the vaccine appeared to prevent or delay recurrences if completed before PSA recurrence in HLA-A2 (+) patients, thereby warranting a larger phase II trial to confirm these findings [174,175]. 

Another example is the AE37 vaccine, which includes an Ii-key-modified HER-2/neu peptide (Ii-key/HER-2 (776–790) or AE37), an immunoregulatory segment of the Ii protein (the Ii-Key peptide) that has been specifically modified so as to loosen the epitope-binding groove of MHC class II molecules and to permit direct charging of MHC class II epitopes to the peptide-binding groove, thereby circumventing the need for intracellular epitope processing [156,171]. The AE37 peptide vaccine has been shown to elicit compelling T helper cell and CTL responses, as well as increased anti-tumor activity, in a series of pre-clinical studies, significantly more so than the native, non-modified HER-2 (776–790) (or AE36) peptide [156,176,177,178]. Following encouraging results from a phase I clinical trial of a hybrid AE37 vaccine (HER2/neu with recombinant GM-CSF as adjuvant) in breast cancer patients [179], the first phase I study testing the same hybrid vaccine in HER2/neu-positive PCa patients demonstrated safety and clinical efficacy in inducing antigen-specific immune responses in patients with castrate-sensitive and castrate-resistant PCa [171]. Immunologic 4-year follow-up assessment revealed vaccine-specific long-term immunity in most patients; notably, those who had received booster vaccination had a more favorable clinical outcome in terms of metastasis-free survival (MFS) or OS as compared to patients with similar clinical characteristics and/or histology at diagnosis who did not receive booster doses, thereby highlighting the need for administering booster shots in order to sustain immunological memory [180]. Furthermore, a retrospective analysis of biomarkers predicting the immunologic and clinical responses to AE37, concluded that patients expressing HLA-A24 and/or HLA-DR11 alleles demonstrate increased vaccination-specific immunity and prolonged OS, as opposed to patients expressing the HLA-A2 allele, who are characterized by high frequencies of circulating Tregs, which is in turn associated with a negative immunological response and decreased OS [181,182]; in addition, lower pre-existing TGF-β plasma levels appear to correlate with a better immunological response to the vaccine and prolonged OS, whereas higher levels of pre-existing IFN-γ-producing T cells are significantly associated with higher delayed-type hypersensitivity (DTH) immune responses and improved OS, as compared to patients with compromised pre-existing immunity to AE36 [183]. Larger cohort studies are warranted in order validate the identified biomarkers and to establish their clinical utility.

## 3. The Main Challenges Associated with the Implementation of Immunotherapy into Clinical Practice and Possible Ways to Circumvent Them 

The implementation of PCa immunotherapy into clinical practice is associated with a number of challenges. First and foremost, the efficacy of immunotherapeutic applications is largely unpredictable; optimal evaluation and timing of vaccine-specific T cell responses remains unclear, despite being considered critical for the selection of therapeutic agents and therefore for the development of precision-tailored therapeutics [50]. Specifically, there is a lack of change in short-term PFS, contrary to what is usually observed with multiple agents [184]. Also, in contrast to cytotoxic chemotherapy agents, tumor burden may not present with significant changes within a short period of time following immunotherapy and seems to rely on immunologic memory [62]. In other words, there is a great need to properly evaluate the correlation of immune response with anti-tumor activity; the incorporation of extended endpoints and additional immune-related criteria in the design of clinical trials, as well as the standardization of immune response methodologies in multicenter trials, may contribute towards a better understanding of its relationship with clinical outcomes [50,185].

Tumor heterogeneity among different patients adds even more complexity to the picture, as it may be driven by far more complex mutational heterogeneity, providing subclonal cell populations with inherent plasticity and the ability to re-differentiate as new clones, thereby contributing to immune resistance and therapeutic failure [185]. This increasing number of genetic mutations detected across tumor types further increases the difficulty to identify clinically significant biomarkers and, subsequently, the patients that are most likely to respond to biomarker-specific treatments. A growing volume of data from clinical trials has demonstrated that only a subset of patients derive clinical benefit from PCa immunotherapy, markedly patients with smaller tumor volumes and less aggressive, or indolent, disease [184,186]. In addition, there is enough evidence to suggest that PCa immunotherapy is more likely to result in improved outcomes if administered as first-line treatment, as for example in the case of Sipuleucel-T or ipilimumab, whereas patients with advanced disease, such as those with lung and liver metastases, may actually fare worse [68,186,187]. It has been suggested that in patients with advanced disease, vaccines may induce an immune reaction in both the normal and tumor prostate tissue and thereby to cause a temporary rise in PSA and in measurable lesions [50]. The latter may be further complicated by the fact that the most common site of metastasis in PCa is the bone which, not only is regarded as a non-measurable site, but it also produces many growth factors and interleukins capable of further stimulating metastatic growth and causing therapeutic resistance [50,188]. In this case, the latter takes place both due to the fact that the oxygen-poor bone microenvironment is rich in Tregs and other tumor suppressor cells, as well as because it releases growth factors, such as TGF-β, that also act to abrogate the immune response [62]. Therefore, immune resistance that occurs before the application of immunotherapy constitutes a major challenge.

The identification of tumor-resistant clones by performing pre- and post-treatment tumor biopsies could aid in identifying the patients most likely to present with acquired immune resistance and in developing biomarker-specific combination treatments to either prevent or bypass therapeutic resistance [185]. Numerous platforms are currently evaluating combinations of treatments, where immunotherapeutic agents are tested both in conjunction with more traditional therapies such as ADT, chemotherapy, and radiotherapy, as well as with other types of immunotherapy; a significant number of these combinations have already been found to augment the effect of immunotherapy, to enhance the anti-tumor immune response and to diminish immune tolerance, leading to improved outcomes [62,184]. In addition, the lack of toxicity that is associated with the majority of the currently investigated immunotherapies, especially vaccines, makes them very attractive candidates for use in both early and adjuvant settings of PCa clinical management; such therapeutic modalities offer the possibility of a longer and unremitting therapeutic response without suffering the unwanted side-effects of toxic treatments, at least so in cases where there is no significant disease progression and toxic treatments can be substantially delayed [116]. In this process, it is imperative to identify additional genetic mutations, biomarkers, and cancer pathways that characterize the pathogenesis of PCa, with the aim to further unravel tumor heterogeneity, to identify clinically significant and targetable tumor antigens and in this way to introduce therapeutic combinations that target multiple mutations at the same time [185]. Liquid and solid tumor biopsies could offer significant advantage in the characterization of tumor heterogeneity and in the identification of the targetable mutations in patients with disease progression; this could lead to more precision-orientated therapeutic decisions and hopefully extend clinical benefit to patient subtypes with more advanced disease [185]. Last but not least, even though the use of immunotherapy for cancer prevention has been largely experimental, the FDA approval of adjuvant immunotherapy for patients with a high risk of melanoma recurrence, offers much hope for the development of similar immunopreventive strategies for other highly immunogenic cancer types, such as PCa [185,189,190]. 

## 4. Conclusions

Immunotherapy has emerged as an attainable and potent weapon in the quiver of precision medicine for the treatment of PCa. Despite the numerous challenges associated with its clinical implementation, immunotherapy constitutes a viable and promising treatment modality, a shift from conventional treatment approaches, that perfectly fits the rationale of precision oncology. The development of new technologies has accelerated the identification of immune-targeted biomarkers with prognostic and predictive significance, largely contributing to the rational, appropriately sequenced combination of treatment schemes and in the matching of patients with targeted therapies so as to achieve the maximum clinical benefit. Even though the majority of patients that seem to derive clinical advantage from the currently available immunotherapeutic strategies markedly fall into localized and/or non-metastatic disease subtypes, there is substantial evidence to suggest that patients with unfavorable characteristics such as predominantly existing metastases may also experience improved outcomes, as seen with cancer vaccines Ad5-PSA and CV9103 and through combinations of ICIs with other treatment modalities. Data from the numerous ongoing clinical trials are expected to shed more light into this rapidly evolving picture of biomarker-tailored immune-mediated therapies, to help us apply immunotherapy to a wider range of PCa patients and to achieve successful treatment even in cases of high-risk or persistent disease. 

## Figures and Tables

**Table 1:** Ongoing clinical trials evaluating immune checkpoint inhibitors (ICIs) as therapeutic regimens for PCa (adapted from Kim et al., 2020 and Rizzo et al., 2020) [59,88].

Agent	CT Phase	Therapeutic Mechanism	Disease Subtype	NCT Identifier	Classification of Evidence *
Atezolizumab +	Ib	IC monotherapy + vaccine	Asymptomatic or minimally symptomatic mCRPC	NCT03024216 [89]	1: Manageable safety profile.
Sipuleucel-T					2: immune responses but no CR
Atezolizumab +	I/II	IC monotherapy + Akt kinase inhibitor	mCRPC with PTEN loss	NCT03673787 [90]	1: Well-tolerated
Ipatasertib					2: early efficacy signals, as evidenced by reductions of Tregs in tumor micro-environment increases in intra-tumoral T cell infiltration,
Avelumab + PT-112	I/II	IC monotherapy + platinum-pyrophosphate conjugate	Advanced mCRPC	NCT03409458 [91]	1: Well-tolerated with evidence of efficacy.
					2: marked therapeutic activity in bone metastases; serologic responses and prolonged disease control in multiple patients.
Avelumab + Talazoparib	II	IC monotherapy + PARP inhibitor	Advanced mCRPC	NCT03330405 [92]	1: Preliminary anti-tumor activity and manageable safety profile.
Durvalumab +	I	IC monotherapy + A2AR antagonist	Advanced mCRPC	NCT02740985 [93]	1: Tolerable with minimal toxicities.
AZD4635					2: associated with clinical benefit, as evidenced by ORR and PSA response rates, as well as baseline TCR diversity and clonality.
Durvalumab +	II	Dual IC blockade	Chemotherapy naïve CRPC	NCT03204812 [94]	0: No evidence has been published yet.
Tremelimumab					
Durvalumab +/-	II	Dual IC blockade	mCRPC	NCT02788773 [95]	1: Insufficient clinical activity as evidenced from ORR.
Tremelimumab					2: Insufficient clinical activity (PSA RR, disease progression, AEs)
Durvalumab +	II	Dual IC blockade + other types of therapy	Biomarker-stratified mCRPC	NCT03385655 [96]	1: Activity in 4 of 7 evaluable cohorts with darolutamide and adavosertib, meeting the requirements for expansion of these arms.
Tremelimumab + Carboplatin or Ipatasertib or Savolitinib or Darolutamide or Adavosertib or CFI-400945					
Ipilimumab + GVAX	I	IC monotherapy + vaccination	mCRPC	NCT01510288 [65]	1: Acceptable safety profile (AEs).
					2: 50% or higher declines in PSA in 25% of patients.
Ipilimumab + Sipuleucel T	I	IC monotherapy + vaccination	Progressive mCRPC	NCT01832870 (SIPIPI) [97]	1: Acceptable safety profile (AEs); antigen-specific anti-tumor responses in chemotherapy-naïve patients.
Ipilimumab	I/II	IC monotherapy + radiotherapy	mCRPC	NCT00323882 [67]	1: manageable AEs and PSA responses suggestive of clinical activity.
					2: Clinical anti-tumor activity with disease control.
Ipilimumab	III	IC monotherapy	mCRPC following docetaxel therapy	NCT00861614	1: No significant difference in OS.
				(CA184-043) [68]	2: PFS significantly superior to OS.
Ipilimumab	III	IC monotherapy	Chemotherapy naïve mCRPC	NCT01057810 [70]	1: No significant imrovement in OS.
					2: Longer median PFS, decline in serum PSA; anti-tumor activity.
Nivolumab	Ib	IC monotherapy	CRPC	NCT00730639	2: No favorable ORR.
				(MDX-1106) [72]	
Nivolumab + Rucaparib	I/II	IC monotherapy + PARP inhibitor	mCRPC	NCT03572478 [98]	0: No evidence has been published yet.
Ipilimumab + nivolumab	II	Dual IC blockade	mCRPC	NCT02985957 (CHECKMATE-650) [99]	2: Superior ORR (26%) in chemo-therapy-naïve patients.
Ipilimumab + nivolumab	II	Dual IC blockade	mCRPC with detectable AR-V7 transcript	NCT02601014	2: More favorable outcomes in patients with AR-V7-positive PCa with DDR.
				(STARVE-PC) [83]	
Pembrolizumab +guadecitabine	I	IC monotherapy + DNA hypo-methylating agent	mCRPC	NCT02998567 [100]	1: No unexpected toxicities.
					2: evidence of TILs suggestive of biological and anti-cancer activity.
Pembrolizumab	Ib	IC monotherapy	Advanced PCa at least 1% PD-L1 expression in tumor or stromal cells	NCT02054806 (KEYNOTE-028) [79]	2: Durable objective response in a subset of patients; favorable side effect profile.
Pembrolizumab + pTVG-HP	I/II	IC monotherapy + vaccine	mCRPC	NCT02499835 [101]	1: Acceptable safety profile.
					2: tumor-targeted T-cell activation.
Pembrolizumab + enzalutamide	Ib/II	IC blockade + ADT	mCRPC	NCT02861573	1: 20% ORR and 33% PSA response rate; sustained activity and safety profile.
				(KEYNOTE-365) [102]	
Pembrolizumab	II	IC monotherapy	Chemotherapy-resistant mCRPC	NCT02787005 (KEYNOTE-199) [80]	2: Substantial anti-tumor activity with an acceptable safety profile in a subset of patients.
Pembrolizumab + Radium-223	II	IC monotherapy + radiotherapy	mCRPC	NCT03093428 [88]	0: No evidence has been published yet.
Pembrolizumab + HER2 BiTEs	II	IC monotherapy + BiTEs	mCRPC	NCT03406858 [103]	1: Well-tolerated with no unexpected toxicities; PFS in a subset of patients.
Pembrolizumab + navarixin (MK-7123)	II	IC monotherapy + CRCX1/CRCX2 antagonist	mCRPC	NCT03473925 [88]	0: No evidence has been published yet.

* Clinical trial classification of evidence: 1 = primary endpoint evidence; 2 = secondary endpoint evidence; 0 = no evidence. A2AR: adenosine 2A receptor; ADT: androgen deprivation therapy; AE: adverse event; AR-V7: androgen receptor variant 7; CT: clinical trial; DDR: DNA damage repair; mCRPC: metastatic castration-resistant prostate cancer; ORR: objective response rate; OS: overall survival; IC: immune checkpoint; ICI: immune checkpoint inhibitors; PARP: poly(ADP-ribose) polymerase; PD-L1: programmed death-ligand 1; PFS: progression-free survival; PSA: prostate-specific antigen; RR: response rate; TCR: T-cell receptor; TILs: tumor-infiltrating lymphocytes; Tregs: T-regulatory cells.

## Abbreviations

AA	African Americans
A2AR	adenosine 2A receptor
Ad5	adenovirus type 5
ADT	androgen deprivation therapy
AdV-tk	adenoviral vector containing a herpes virus-derived thymidine-kinase gene
AE	adverse event
AR	androgen receptor
AR-V7	androgen receptor variant 7
APC	antigen-presenting cell
CA	Caucasian Americans
CEA	carcinoembryonic antigen
ChAd	chimpanzee adenovirus
CRPC	castrate-resistant prostate cancer
CT	clinical trial
CTLA-4	cytotoxic T-lymphocyte associated antigen 4
CTL	cytotoxic T-lymphocyte
DC	dendritic cell
DDR	DNA damage repair
DTH	delayed-type hypersensitivity
EMP	estramustine phosphate
GM-CSF	granulocyte macrophage-colony-stimulating factor
HER2	human epidermal growth factor receptor 2
HLA	human leukocyte antigen
IC	immune checkpoint
ICAM-1	intracellular adhesion molecule-1
ICI	immune checkpoint inhibitors
IFN	interferon
LLO	listeria monocytogenes (Lm)-listeriolysin O
LNCaP	lymph node-derived human prostate adenocarcinoma cell line
mAbs	monoclonal antibodies
mCRPC	metastatic castration-resistant prostate cancer
MHC	major histocompatibility complex
MFS	metastasis-free survival
MRM	mismatch repair mechanism
MSI	microsatellite instability
MUC1	mucin-1
ORR	objective response rate
OS	overall survival
PAP	prostatic acid phosphatase
PARP	poly(ADP-ribose) polymerase
PCa	prostate cancer
PCA3	prostate cancer antigen 3
PC-3	prostate cancer cell line derived from bone metastasis
PD-1	programmed death receptor-1
PD-L1	programmed death-ligand 1
PPV	personalized peptide vaccination
PSA	prostate-specific antigen
PSCA	prostate stem cell antigen
PSMA	prostate-specific membrane antigen
scFv	single chain variable fragment
STEAP	six transmembrane epithelial antigen of the prostate
TCR	T cell receptor
TAAs	tumor-associated antigens
TARP	T-cell receptor alternate reading frame protein
TCR	T cell receptor
TGF-β	tumor growth factor β
TILs	tumor infiltrating lymphocytes
TMB	tumor mutation burden
TRICOM	TRIad of CO-stimulatory Molecules
